# Polymyxin B-immobilized fiber hemoperfusion therapy improves sepsis-related immunosuppression

**DOI:** 10.1186/cc13597

**Published:** 2014-03-17

**Authors:** A Kimura, S Ono, S Hiraki, R Takahata, H Tsujimoto, M Kinoshita, S Aosasa, K Hatsuse, D Saitoh, K Hase, J Yamamoto

**Affiliations:** 1 National Defense Medical College, Tokorozawa, Japan

## Introduction

Sepsis-induced immunosuppression has long been considered a factor in the late mortality of sepsis patients, but little is known about the immunity of immunocompetent cells and the effect of polymyxin B-immobilized fiber hemoperfusion therapy (PMX- DHP) on sepsis-induced immunosuppression. The present study was designed to evaluate the effect of PMX-DHP on recovery from sepsis- related immunodeficiency.

## Methods

Patients with septic shock who were treated with PMX- DHP were enrolled in this study. Study 1: (1) numbers of peripheral lymphocytes and CD4+ T cells, especially regulatory T cells (Tregs), and serum cytokine levels were examined to evaluate the effects of PMX- DHP in septic shock patients. (2) Peripheral blood mononuclear cells (PBMCs) in these patients were examined to evaluate inflammatory cytokine production before and after PMX-DHP. The obtained PBMCs were stimulated with interleukin (IL)-2 and IL-12, anti-CD3 antibody, or lipopolysaccharide for 24 hours, and tumor necrosis factor alpha and interferon-gamma (IFNy) production in the culture supernatants was measured using enzyme-linked immunosorbent assay. Study 2: whole blood from patients with sepsis was incubated with a polymyxin B-immobilized filter (cut into small sizes) for small animals for 2 hours (PMX group), or were treated with 200 μg polymyxin B for 2 hours (PLB group), or were not treated (sepsis group). IFNy production by PBMCs was compared among the three groups.

## Results

Study 1: (1) the number of CD4+ T cells was lower and the percentage of Tregs in CD4+ T cells was higher in septic shock patients compared with those without shock. A significant increase in the number of CD4+ T cells, a significant decrease in the percentage of Tregs in the CD4+ T-cell population, and a significant decrease in serum IL-10 levels were observed 24 hours after PMX-DHP in septic shock patients who survived compared with those who did not. (2) IFNy production by PBMCs was significantly lower in patients with sepsis than in healthy volunteers. IFNy production by IL-2-stimulated and IL-12-stimulated PBMCs significantly increased after PMX-DHP therapy. Study 2: IFNy production by PBMCs in patients with sepsis increased significantly in the PMX and PLB groups compared with that in the sepsis group.

## Conclusion

PMX-DHP directly decreased the number and percentage of Tregs in peripheral blood circulating CD4+ T cells in patients with septic shock. PMX-DHP improved IFNy production by natural killer (NK)/NKT cells in patients with septic shock. Therefore, PmX-DHP could improve sepsis-related immunosuppression.

